# Application of Mini-LEDs with Microlens Arrays and Quantum Dot Film as Extra-Thin, Large-Area, and High-Luminance Backlight

**DOI:** 10.3390/nano12061032

**Published:** 2022-03-21

**Authors:** Yen Lung Chen, Zhi Ting Ye, Wei Lai, Chang Che Chiu, Kuo Wei Lin, Pin Han

**Affiliations:** 1 Graduate Institute of Precision Engineering, National Chung Hsing University, Taichung 402, Taiwan; d105067001@mail.nchu.edu.tw; 2 Darwin Precisions Corporation, Hukou Township, Hsinchu City 300, Taiwan; 3 Department of Mechanical Engineering, Advanced Institute of Manufacturing with High-Tech Innovations, National Chung Cheng University, Chia-Yi 62102, Taiwan; william19961758@gmail.com (W.L.); g10421033@ccu.edu.tw (C.C.C.); 4 Metal Industries Research & Development Center, Kaohsiung City 800, Taiwan; linkuowei@mail.mirdc.org.tw

**Keywords:** mini-light-emitting diodes, extra-thin, high luminance, microlens arrays (MLAs), quantum-dot film, uniformity merit function, total internal reflection

## Abstract

The demand for extra-thin, large-area, and high-luminance flat-panel displays continues to grow, especially for portable displays such as gaming laptops and automotive displays. In this paper, we propose a design that includes a light guide layer with a microstructure above the mini-light-emitting diode light board. The light control microstructure of concave parabel-surface microlens arrays on a light-emitting surface increases the likelihood of total internal reflection occurring and improved the uniformity merit function. We used a 17 in prototype with quantum-dot and optical films to conduct our experiments, which revealed that the thickness of the module was only 1.98 mm. When the input power was 28.34 watts, the uniformity, average luminance, and CIE 1931 color space NTSC of the prototype reached 85%, 17,574 cd/m^2^, and 105.37%, respectively. This module provided a flat light source that was extra thin and had high luminance and uniformity.

## 1. Introduction

Display technology is widely used in our daily lives in display applications such as computer monitors, televisions, augmented/virtual reality devices, and smartphones. With improvements in living standards, the requirement for high-end displays is also increasing, and the demand for features, such as thinness, high luminance, high color saturation, and high contrast, is increasing [1,2,3,4]. With the advancement of new technologies, traditional liquid crystal display (LCD) technologies are gradually being surpassed by novel display technologies, such as organic light-emitting diodes (OLEDs) and quantum-dot light-emitting diodes (QLEDs), in terms of contrast, color gamut, and brightness [5,6,7,8]. OLED technology provides a wide color gamut, bright colors, flexible shapes, and an excellent black area that can be completely nonemitting. However, its disadvantages include rapid material aging, screen burn-in, and a luminance level that is difficult to increase to more than 1000 nits [9,10,11]. By contrast, LCDs have low cost, long service life, and low power consumption. LCDs are still the mainstream display technology in the current market. However, LCDs have a low contrast ratio and low photoelectric conversion efficiency [12,13,14]. At present, blue-light GaN-based light-emitting diodes (LEDs) are used as the backlight module of LCD light sources, and they serve the purpose of exciting yellow phosphor to form a white light source. However, phosphors have disadvantages such as low efficiency, a wide emission spectrum, and poor particle uniformity. Moreover, the lack of the red-light band in their emission spectrum leads to poor color rendering. A method for improving color saturation is to add green and red phosphors to yellow phosphors [15,16,17]. Quantum dots (QDs), which are a new type of color conversion material, features narrow emission spectrum, tunability, and high color purity. An emerging practice in the field of display technology is the application of a QLED coating to LEDs, which was started to be used in backlight modules [18,19,20]. In recent years, the demand for high dynamic range (HDR), low power consumption, and thinness at the application level of high-end displays has led to the use of mini-LEDs and micro-LEDs as backlight sources for LCDs [21,22,23]. Mini-LEDs and micro-LEDs have smaller package sizes than traditional LEDs. They also allow for higher current densities and meet the requirements for thinness and lightness. Micro-LEDs have high contrast ratios, low delay times, and high color saturation, but their yield and mass transfer are suboptimal [24,25,26]. When mini-LEDs are used as light sources for backlight modules to achieve HDR control with local dimming, high contrast and low power consumption can be achieved [27,28,29]. Direct-lit backlight modules provide high optical efficiency, achieve local dimming with ease, and have excellent HDR performance. Therefore, mini-LED backlight modules are typically used in direct-lit backlight modules [30,31,32]. Generally, the light angle of mini-LEDs in the package form is 120°. Because a mini-LED is smaller in size than a traditional LED, more mini-LEDs must be used to achieve high uniformity. However, this increase in the number of LEDs results in an over concentrated heat source. Moreover, halo effects can emerge if no adjustments for HDR are made [33,34,35]. Several studies have used optical design to optimize the light-emitting angle of mini-LEDs; this approach can reduce the number of LEDs used and improve uniformity [36,37]. Other studies have also been conducted in this field. Tang et al. proposed the use of photonic crystals as a spatially periodic refractive index material for controlling the direction of light, improving the light extraction efficiency and uniformity of LEDs, and enhancing the performance of mini-LEDs [38]. Jiao et al. proposed the use of InGaN/GaN nanorod LED arrays with nanoimprint and reactive ion etching to increase the internal quantum efficiency and light extraction efficiency of LEDs [39]. Ye et al. proposed the deposition of high-reflectivity thin films on chip surfaces to improve light angles [40]. Zhu et al. proposed a diffused transmission freeform surface for LED illumination that increases illumination efficiency and provides high uniformity [41]. Sun et al. proposed a total-internal-reflection structure that is based on the ray-mapping method and provides improved light extraction efficiency and uniformity [42]. Ye et al. proposed a fully printed diffuse reflection design and a dotless light guide plate design for the bottom section of a light source module; these designs provide high uniformity and efficiency [43]. Ye et al. have also proposed a hollow light guide that reduces weight, increases efficiency, and uses multiple angles on the end wall of a module to reflect light such that high uniformity is achieved [44]. Lu et al. proposed the placement of 32 × 32 mini-LEDs on a polyethylene terephthalate-based, transparent, and flexible substrate to achieve high uniformity and a wider color gamut [45]. Chen et al. proposed a new type of packaging technology for direct-lit micro-LEDs (mini-CSPLEDs) that combines a QD film and a diffuser to achieve high uniformity and increase emission angles. When the full angle of light is 120°, the uniformity merit function (UMF) pitch/optical distance (OD) is approximately between 0.8 and 1.0; when it is 180°, the UMF pitch/OD increases to 2.04 [46]. Feng et al. proposed a mini-LED backlight module that uses an optical film with microstructures that are smaller than mini-LEDs to improve uniformity without the use of precise positioning [47]. The aforementioned optical designs focus on increasing light angle, color saturation, uniformity, and light output efficiency. In their research on module thinning, Ye et al. proposed the use of mini-LEDs that incorporate multiple three-dimensional (3D) diffuse reflection cavity arrays to develop a design that enables thin backlight modules to achieve high uniformity and luminance [48]. Similarly, Kikuchi et al. proposed a mini-LED backlight module with highly reflective mirror dots, which is a design that utilizes the structure of mirror dots and the slot between LEDs to improve uniformity and suppress the halo effect; although the thickness of the module was successfully reduced, its light extraction efficiency was only 58.8% [49]. In conclusion, few studies have examined extra-thin, large-area, high-luminance, and high-uniformity properties. Therefore, we propose a design that includes a light guide layer above the mini-LED light board and microlens arrays (MLAs) on the light-emitting surface. The light control microstructure of concave parabel-surface MLAs (PSMLAs) increases the UMF. The design provides a large-area flat light source that is extra-thin and achieves high uniformity and luminance. This design has high potential applicability in high-end displays.

## 2. Methods

### 2.1. Simulation of Mini-LEDs with an MLA Unit Module and a Light Film Material Selection

**Figure 1:**
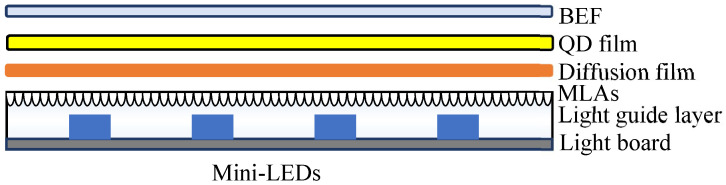
Structure of an extra-thin, large-area, high-luminance surface light source module with microlens arrays (MLAs) combined with a light guide layer. BEF, brightness enhancement film; QD, quantum dot; LED, light-emitting diode.

We used the 3D drawing software SolidWorks (Dassault Systèmes, Vélizy-Villacoublay, France) and the optical simulation software LightTools (Synopsys, Mountain View, CA, USA) to optimize the design of extra-thin, large-area, high-luminance surface light-source modules. The optical components comprise mini-LEDs arrays, a light board, light guide layers combined with MLAs, a diffusion film, a QD film, and a brightness enhancement film (BEF). The schematic of the extra-thin, large-area, high-luminance surface light source module with MLAs combined with the light guide layer is presented in Figure 1.

**Figure 2:**
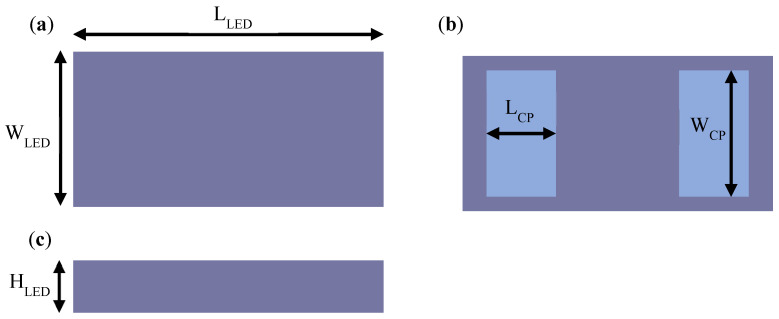
Structure of mini-LEDs: (**a**) top view; (**b**) bottom view; (**c**) side view. Adapted from ref. [48].

The length (L_LED_), width (W_LED_), and height (H_LED_) of the mini-LEDs were 320, 210, and 100 μm, respectively. The length (L_CP_) and width (W_CP_) of the chip pad were 75 and 170 μm, respectively. The package size of the mini-LEDs is illustrated in Figure 2.

**Figure 3:**
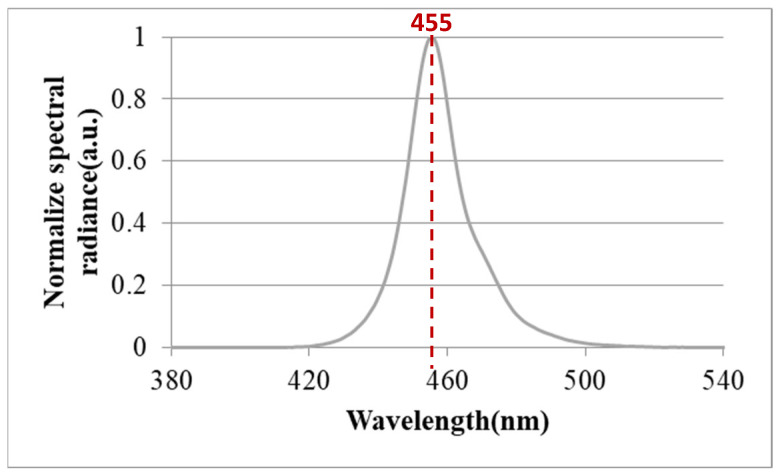
Normalized electroluminescence spectrum of mini-LEDs.

The normalized electroluminescence (EL) spectrum of the mini-LEDs is plotted in Figure 3. The peak wavelength of the light source was 455 nm.

**Figure 4:**
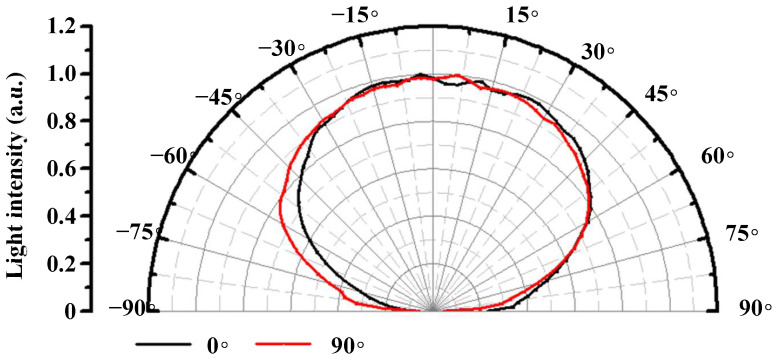
Mini-LED chip light distribution curves.

The light distribution curve of the mini-LEDs is plotted in Figure 4. In the figure, the full width at half maximum (FWHM) of the light angle was 140°, and the black and red curves represent the light distribution curves of the horizontal and vertical sections, respectively.

**Figure 5:**
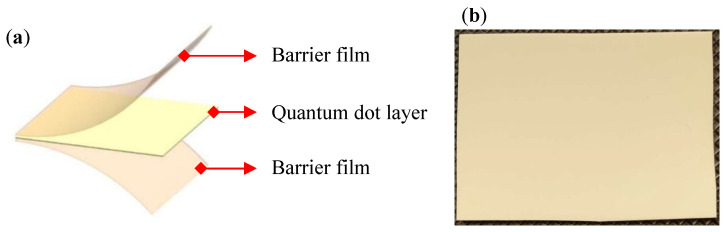
CdSe quantum-dot film: (**a**) schematic structure; (**b**) prototype.

The QC100B QD film (UBright Optronics Corporation, Taoyuan City, Taiwan) was used (its structure is illustrated in Figure 5). The QD’s film thickness was 100 μm, and its applicable excitation wavelength was between 448 nm and 455 nm; the peak wavelength and FWHM of the spectrum for the green QD were 534 nm and 23 nm, respectively; the peak wavelength and FWHM of the spectrum for the red QD were 629 nm and 23 nm, respectively. According to the EL spectrum in the CIE 1931 color gamut coordinates, the part-to-part white point variation of the film was ≤0.01 for both x and y.

### 2.2. Model Construction of the MLA Unit

We propose the incorporation of a light guide layer in the design of an MLA microstructure array to improve the UMF. The theory of total reflection suggests that the light of mini-LEDs can be guided to the lateral surface through the MLA structure to expand its light output range.

**Figure 6:**
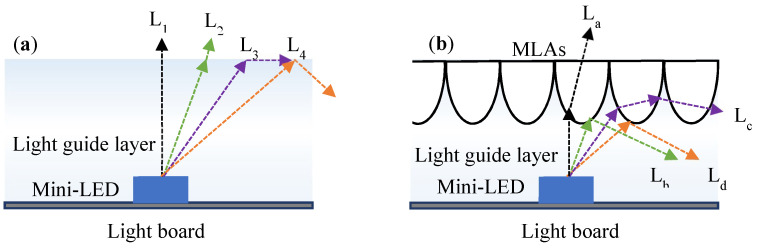
Ray tracing of the light guide layer combined with the MLA module: (**a**) ray tracing without MLAs; (**b**) ray tracing with MLAs.

Figure 6a presents a design without MLAs. After passing through the light guide layer, the light from L_1_–L_2_ directly exits the light guide layer because it is not greater than the total reflection angle. L_2_ is the light that satisfies the total reflection angle, and L_3_ is the light that is greater than the total reflection angle; therefore, the light is fully internally reflected. Figure 6b presents the structure of the light guide layer that is combined with MLAs. After passing through the MLAs, the light of L_a_ directly exits the light guide layer, and the light of L_b_ and L_d_ is the light with angles that are greater than the total reflection angle, and this light is fully internally reflected. Light from L_c_ is refracted onto adjacent microstructures. The combination of the MLA microstructure with the light guide layer results in a wider light-emitting area for a given thickness and reduced use of mini-LEDs for improving the UMF.

The microstructure of the MLAs has a quadratic surface design, and the optimal curvature was calculated using the quadratic Equation (1) as follows:

(1) $$Z\left(r\right)=\frac{{\mathrm{cr}}^{2}}{1+\sqrt{1-\left(1+k\right)c^{2}r^{2}}}$$

where c is the curvature, the radius, r, is the reciprocal of the curvature, c=1r, and k is the conic constant, with different conic constants representing different surface types.

**Figure 7:**
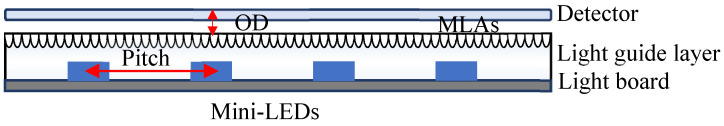
Calculation of the uniformity merit function. OD, optical distance.

Figure 7 is a schematic of the UMF. Numerous mini-LEDs arrays were arranged on the light board, and the light source was guided to both sides of the light guide layer through the combination of the light guide layer with the MLA microstructure design, thus reducing the quantity of mini-LEDs that were used to achieve a thinner and lighter module. The distance between the two adjacent centers of the mini-LEDs was indicated by pitch, and the distance from the top of the MLAs to the detector was indicated by the OD. The UMF was used to determine the association among the thickness, uniformity, and mini-LED pitch of the surface light source module.

For a surface light source with a given area, a greater UMF value indicates a thinner module and a lower number of mini-LEDs being used. The UMF Formula (2) is as follows.

(2) $$\mathrm{Uniformity}\;\mathrm{merit}\;\mathrm{function}\;\left(\mathrm{UMF}\right)=\frac{\mathrm{Pitch}\;\left(\mathrm{mm}\right)\;}{\mathrm{OD}\;\left(\mathrm{mm}\right)}$$

For parameter settings, the refractive index of cross BEF and BEF was set to 1.56, and the vertex angle was set to 90°. The light guide layer was made of poly(methyl methacrylate), and its refractive index was 1.5. The light board surface was a Lambertian diffuse surface with a reflectivity of 90%. The center wavelength of the light source was 450 nm, the output power was normalized to 1 W, and 50 million rays were used for the simulation. The parameter settings for the simulation are listed in Table 1.

**Figure 8:**
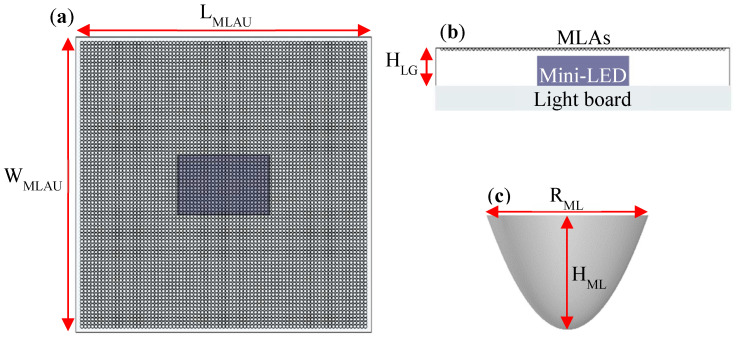
3D structure of the MLA unit module: (**a**) top view; (**b**) side view; (**c**) single microstructure of the MLA.

The light source mini-LEDs are bonded onto the surface of the light board, and a light guide layer was attached to the light-emitting surface of the mini-LEDs. The light-emitting surface of the light guide layer was formed by numerous quadric surfaces that constitute an MLA module. To simplify the simulation settings and optimize the simulation design, the length (L_MLAU_), width (W_MLAU_), and thickness (H_LG_) of the light guide layer of each MLA unit module were set to 1, 1, and 0.25 mm, respectively. The related 3D structure is illustrated in Figure 8a,b, which present the top and side views, respectively, of the 3D module. The diameter and height of the MLA microstructure model are represented by RML and HML, respectively, and its 3D structure is illustrated in Figure 8c.

### 2.3. Simulation and Optimization of the MLA Unit

**Figure 9:**
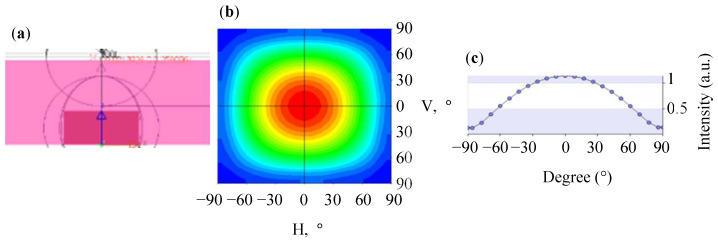
Simulation of extra-thin surface light guide layer without MLAs: (**a**) 3D-simulated structure diagram; (**b**) plane rectangular light distribution curve; (**c**) horizontal section light distribution curve.

First, we simulated the light distribution curve of the extra-thin surface light guide layer without an MLA microstructure. Figure 9a presents the 3D simulation structure in which no MLA microstructure was used on the light guide layer. Figure 9b presents a plane rectangular light distribution of the simulation. The average FWHM of the light angle was 117.57°, and the horizontal section light distribution curve is plotted in Figure 9c. The results indicate that the energy of the light source was excessively concentrated in the center. This problem limits the light output range of mini-LEDs such that the UMF cannot be improved.

**Figure 10:**
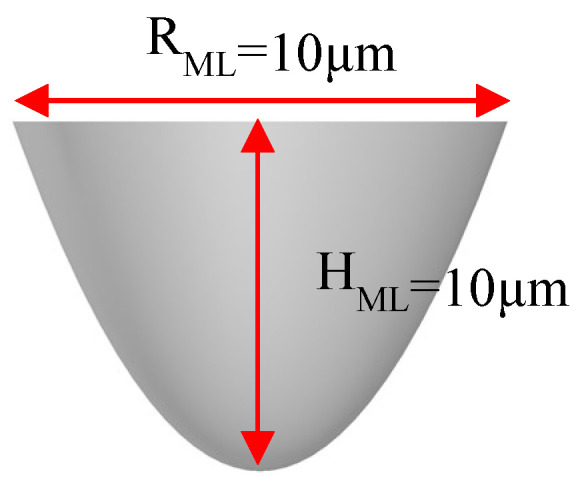
Parabel-surface MLA (PSMLA) microstructure.

We identified the optimal curvature after optimizing Equation (1). The PSMLA microstructure had a conic constant K of −1, texture height H_ML_ of 10 μm, and a texture diameter R_ML_ of 10 μm (Figure 10).

The distance between the upper and lower PSMLAs (X_pitch_) and the left and right PSMLAs (Y_pitch_) was 15 and 15 μm, respectively. The cover rate was calculated using Formula (3), which is as follows:

(3) CoverRate=πr212×Xpitch×Ypitch

where r is the radius of the PSMLA. According to the parameters, the cover rate of PSMLAs was 69.8%.

**Figure 11:**
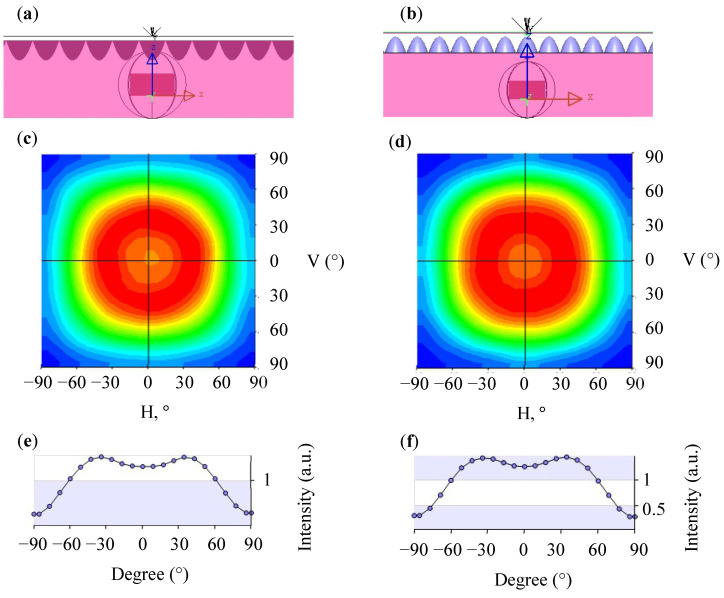
Simulation of the light guide layer combined with concave and convex PSMLA microstructures: (**a**) 3D-simulated structure of concave PSMLAs; (**b**) 3D-simulated structure of the convex PSMLAs; (**c**) plane rectangular light distribution curve of concave PSMLAs; (**d**) plane rectangular light distribution curve of convex PSMLAs; (**e**) horizontal section light distribution curve of concave PSMLAs; (**f**) horizontal section light distribution curve of convex PSMLAs.

Figure 11 presents the simulation results of the light guide layer with the concave and convex PSMLA microstructures. Figure 11a,c are 3D simulation structure diagrams. Figure 11b,d plot the light distribution curves of the simulation and indicate a further expansion of the FWHM of the light angle to 141.82° and 141.45°, respectively. The related horizontal section light distribution curves are plotted in Figure 11e,f.

The aforementioned simulation data are summarized in Table 2. The data reveal that a more favorable light exit angle can be obtained by combining the light guide layer with optimized concave PSMLAs. The addition of a microstructure increased the FWHM of the light angle by 24.25°. Therefore, we adopted this microstructure design to improve the light distribution range of the mini-LEDs array, further improve the UMF, and develop a surface light source module that was extra-thin and had a large area and high uniformity.

**Figure 12:**
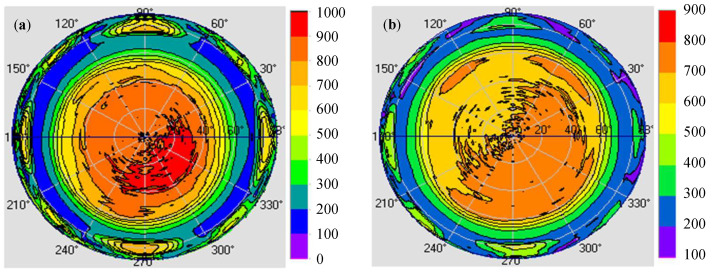
Simulation of the light guide layer unit module with a diffusion film: (**a**) without concave PSMLAs; (**b**) with concave PSMLAs.

After completing the design of the microstructure, we examined the influence of the microstructure on the backlight module. Figure 12 presents the simulation results of the light guide layer unit module that was combined with a diffusion film. Figure 12a illustrates the light polar diagram of the light guide layer that was combined with the diffusion film without a concave PSMLA microstructure. After the uniformity was adjusted using the diffusion film, the FWHM of the light angle was 113°. Figure 12b illustrates the light polar diagram of the light guide layer that was combined with a concave PSMLA microstructure and a diffusion film, and the FWHM of the light angle was 130°. A comparison of the aforementioned simulation data revealed that the difference in microstructures could expand the FWHM of the light angle by 17°, thereby improving its light distribution range and mitigating the excessive concentration of the light source energy in the center.

**Figure 13:**
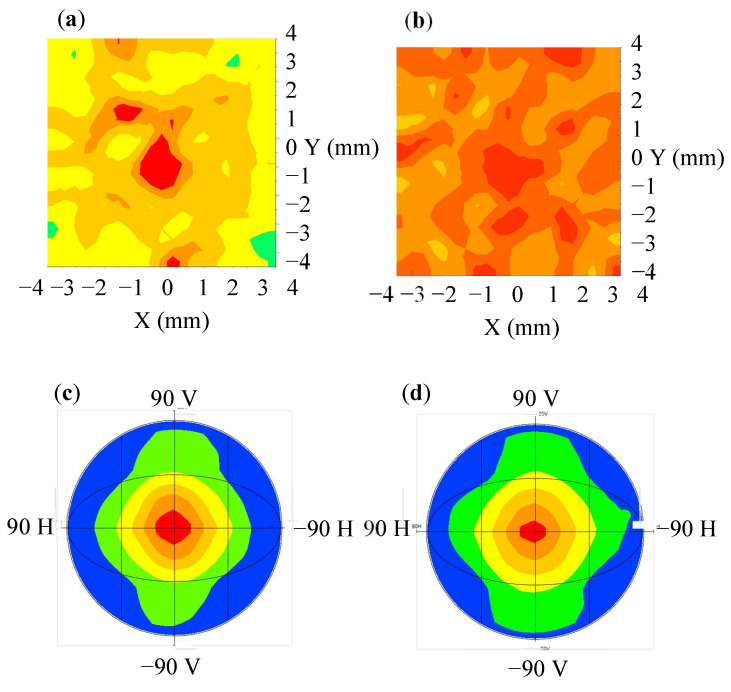
Simulation of an extra-thin, large-area, high-luminance surface light source backlight unit (BLU): (**a**) luminance distribution of a BLU without concave PSMLAs; (**b**) luminance distribution of a BLU with concave PSMLAs; (**c**) light polar diagram of a BLU without concave PSMLAs; (**d**) light polar diagram of a BLU with concave PSMLAs.

Figure 13 presents the simulation results of the light guide layer unit module that was combined with a diffusion film, QD film, and BEF to form an extra-thin, large-area, high-luminance surface light source backlight unit (BLU). Figure 13a presents the extra-thin, large-area, high-luminance surface light source BLU without concave PSMLAs; the average luminance, central luminance, and uniformity were 17,705 nits, 21,117 nits, and 61%, respectively. Figure 13b presents the extra-thin, large-area, high-luminance surface light source BLU combined with concave PSMLAs; the average luminance, central luminance, and uniformity were 17,822 nits, 18,006 nits, and 85.1%, respectively. The results indicate that the addition of a concave PSMLA structure increased the uniformity by 24.1%. Figure 13c,d are light polar diagrams of the extra-thin, large-area, high-luminance surface light source BLU. After the light converged, and the luminance was further improved, the FWHM of the light angle was 51°. This result indicates that the presence or absence of concave PSMLAs did not affect the view angle performance of the module.

## 3. Results and Discussion

**Figure 14:**
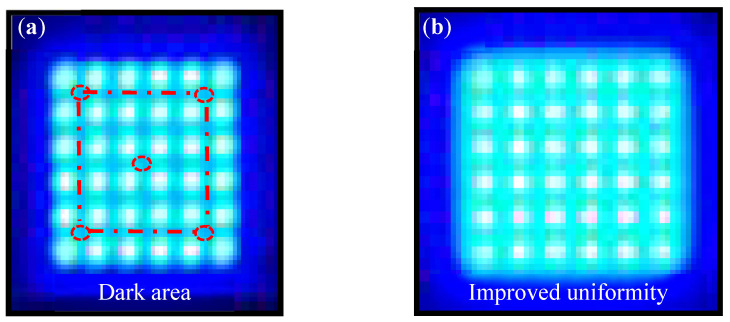
Samples of the light guide layer unit module: (**a**) without PSMLAs; (**b**) with concave PSMLAs.

Figure 14 presents a sample of the light guide layer unit module. Figure 14a shows the light guide layer unit module without concave PSMLAs, and noticeable dark areas are present between the mini-LEDs. Figure 14b displays the light guide layer unit module that was combined with the concave PSMLAs, which considerably reduced the dark areas between the mini-LEDs.

**Figure 15:**
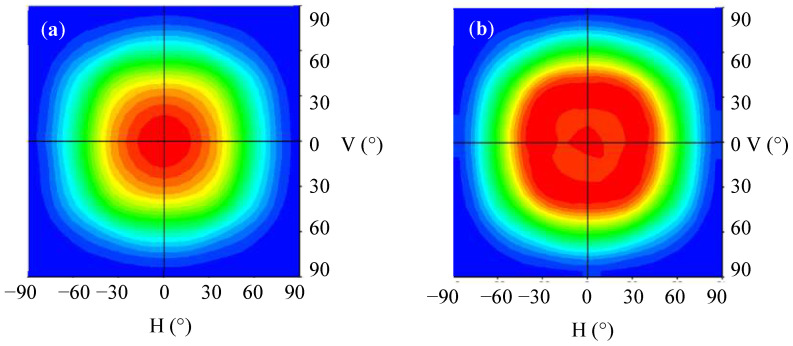
Plane rectangular light distribution curves of the light guide layer unit module combined with a diffusion film: (**a**) without concave PSMLAs; (**b**) with concave PSMLAs.

Figure 15 plots the measured plane rectangular light distribution curves of the light guide layer unit module that was combined with a diffusion film. Figure 15a presents the results for the diffusion film that was combined with the light guide layer unit module without concave PSMLAs for which the FWHM of the light angle was 113°. Figure 15b shows the curve of the light guide layer unit module that was combined with a concave PSMLA structure and a diffusion film, with the FWHM of the light angle increased to 130°. The results indicate that after the adjustment of the microstructure, the light source energy concentrated in the center diffused, and the distribution range of the light emitted by the mini-LEDs could be expanded.

**Figure 16:**
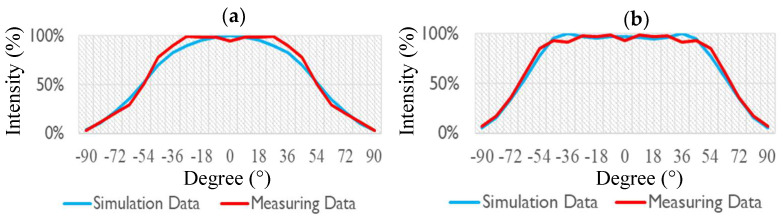
Light distribution curves of the light guide layer unit module combined with diffusion film (simulation optimization and measurement results): (**a**) without concave PSMLAs; (**b**) with concave PSMLAs.

Figure 16 plots the light distribution curves of the light guide layer unit module that was combined with a diffusion film and reveals the simulation optimization and measurement comparison results. Figure 16a presents the results for the diffusion film that was combined with the light guide layer unit without concave PSMLAs. Figure 16b presents the results for the light guide layer unit module that was combined with a concave PSMLA structure and a diffusion film. The blue line represents the simulation results, whereas the red line represents the measurements taken from the sample. A comparison of the simulated and measure data reveals that they were similar.

**Figure 17:**
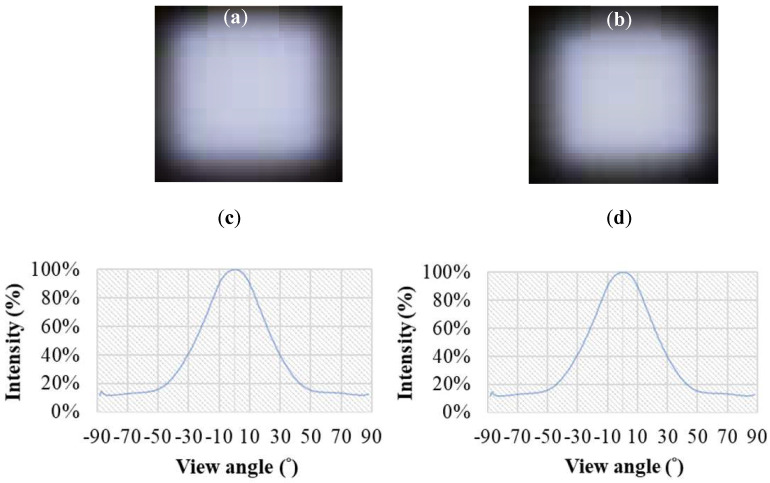
Lit-up sample of extra-thin, large-area, high-luminance surface light source BLU module: (**a**) without concave PSMLAs; (**b**) with concave PSMLAs; (**c**) light distribution curve without concave PSMLAs; (**d**) light distribution curve with concave PSMLAs.

Figure 17 shows a sample of the extra-thin, large-area, high-luminance surface light source BLU module. Figure 17a shows the surface light source BLU module without concave PSMLAs; its light angle is plotted in Figure 17c, and the FWHM of the light angle was 51°. Figure 17b shows the extra-thin, large-area, high-luminance surface light source BLU module that was combined with a concave PSMLA structure; its measured light angle is plotted in Figure 17d, and the FWHM of the light angle was 51°, which is a distribution that was similar to that of the module without PSMLAs. This finding indicates that the presence or absence of concave PSMLAs did not affect the view angle performance of the module.

**Figure 18:**
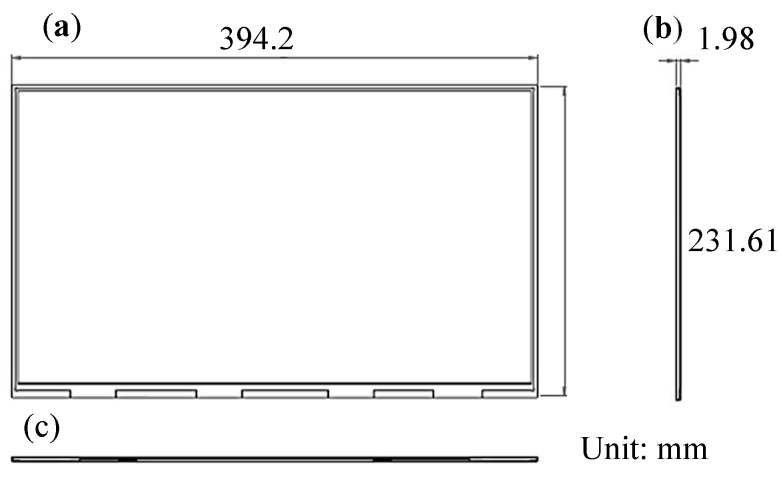
Three-view drawing of 17 in extra-thin, large-area, high-luminance surface light source module: (**a**) top view; (**b**) side view; (**c**) front view.

Figure 18 illustrates the dimensions of the 17 in extra-thin, large-area, high-luminance surface light source module. The module comprised a 0.4 mm printed circuit board substrate; 3024 sets of concave PSMLAs light guide layer unit module (pitch of the length (*x*-direction), 5.3 mm; pitch of the width (*y*-direction), 5.1 mm; OD, 0.4 mm; UMF for length, 4.49; UMF for width, 4.32) that consisted of a light guide layer (thickness: 0.25 mm) and concave PSMLAs (thickness: 0.15 mm); a 0.45 mm diffusion plate with a QD film, diffusion film, and cross BEF optical film. The length, width, and thickness of the module were 394.2, 231.61, and 1.98 mm, respectively. A total of 3024 mini-LEDs were used in the module.

**Figure 19:**
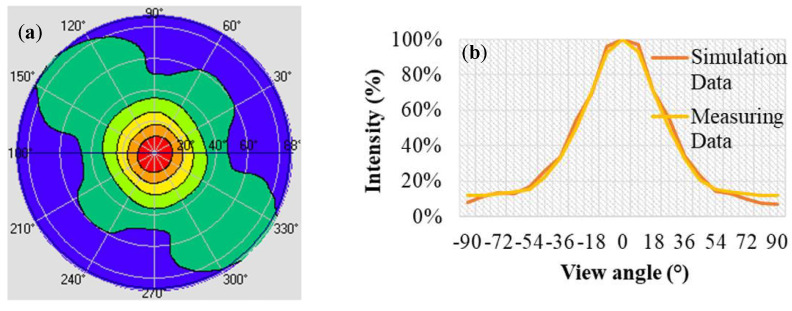
Light distribution of extra-thin, large-area, high-luminance surface light source module: (**a**) light polar diagram of measured data; (**b**) comparison of light distribution curves of the simulation and measurement results.

Figure 19 plots the simulation results and measurements of the 17 inch extra-thin, large-area, high-luminance surface light source module. Figure 19a presents the light polar diagram of the module. Figure 19b presents the light distribution curves of the simulation and measurement results; a comparison revealed that these results were almost identical; for the module, the FWHM of the light angle was 51°, which is consistent with the simulation’s optimization results. Therefore, the design and optimization of the extra-thin, large-area, high-luminance surface light source module was complete.

**Figure 20:**
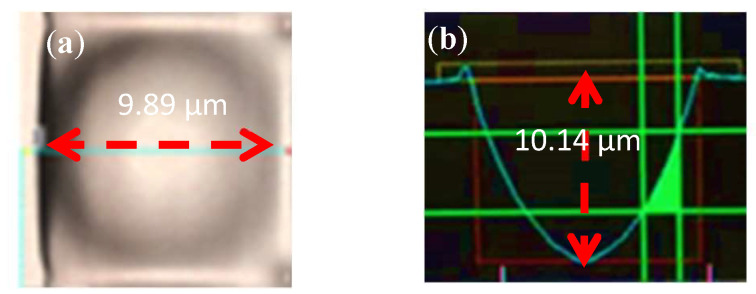
Sectional measurement of a concave PSMLA microstructure sample: (**a**) top view; (**b**) side view.

Figure 20 presents the sectional measurements of the concave PSMLA microstructure sample. Figure 20a,b show the top and side views, respectively. We used KEYENCE VK-9510 (Keyence Corporation, Osaka, Japan) to measure the concave PSMLA microstructure sample. The section diameter and depth of the sample were 9.89 and 10.14 μm, respectively.

**Figure 21:**
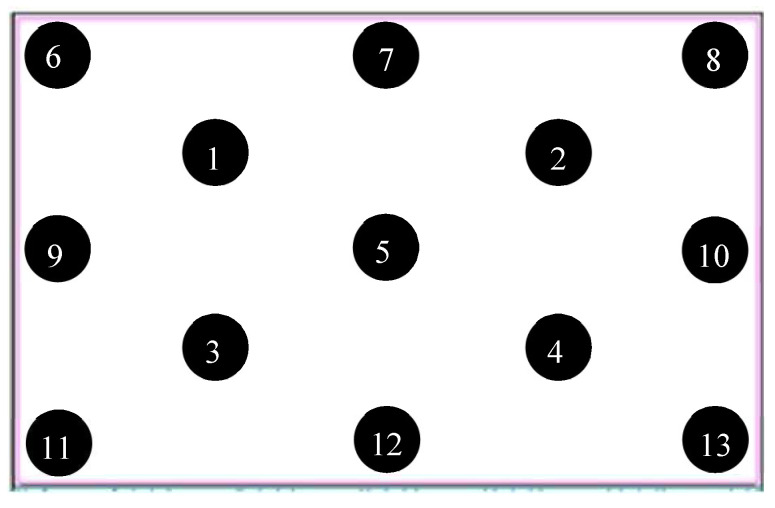
Thirteen-point uniformity measurement of the 17 in extra-thin, large-area, high-luminance backlight module.

Figure 21 illustrates the schematic of the 13 point uniformity measurement of the 17 in extra-thin, large-area, high-luminance backlight module.

Thirteen-point uniformity was calculated using Formula (4), which was as follows:

(4) $$\mathrm{Uniformity}\;(\%)=100\%\;\times \;\frac{\mathrm{minimum}\;\mathrm{luminance}\;\left(\mathrm{nits}\right)}{\mathrm{maximum}\;\mathrm{luminance}\;\left(\mathrm{nits}\right)}$$

The 13 point uniformity measurement was taken when the input voltage, total input current, and total input power of the 17 in extra-thin, large-area, high-luminance light source module were 11.04 V, 2.57 A, and 28.34 W, respectively. The results are presented in Table 3. The average luminance, central luminance, and uniformity of the module were 17,574 nits, 18,852 nits, and 85.48%, respectively.

**Figure 22:**
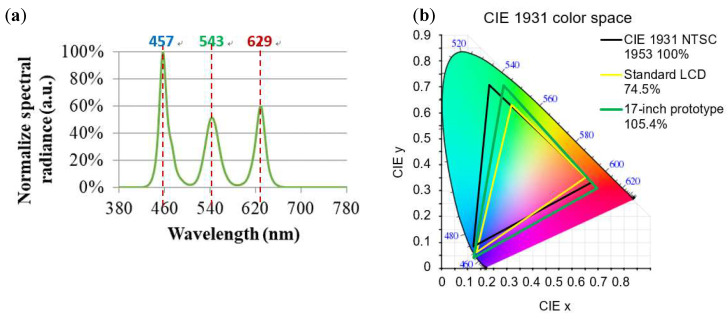
(**a**) Normalized emission spectrum and (**b**) color gamut of an extra-thin, large-area, high-luminance surface light source module.

Figure 22 plots the normalized emission spectrum and color gamut of the 17 in extra-thin, large-area, high-luminance surface light source module. Figure 22a reveals that the peaks of its emission wavelengths were at 457, 543, and 629 nm. Figure 22b plots the color gamut measurement values of the module and reveals that its CIE 1931 color space reached 105.37%, which is an extremely wide color gamut range.

Table 4 lists the color gamut coordinate parameters of the 17 in extra-thin, large-area, high-luminance surface light source module. The coordinate position (*x*, *y*) in the CIE 1931 color gamut coordinate was determined according to the emission spectrum of the module.

## 4. Conclusions

We proposed an optimized design of a light guide layer with concave PSMLAs that used mini-LEDs as the light source for extra-thin, large-area, flat backlight modules. We used a 17 in prototype for the experiments. The thickness of the module was only 1.98 mm. For the mini-LEDs, its pitches in the *x*- and *y*-directions were 5.3 and 5.1 mm, respectively, and its UMFs in the *x*- and *y*-directions were 4.49 and 4.32, respectively. When the input power was 28.34 W, the uniformity, average luminance, and CIE 1931 color space NTSC reached 85%, 17,574 cd/m^2^, and 105.37%, respectively; thus, the flat light source module was extra-thin and provided high uniformity and luminance.

In the future, extra-thin, high-luminance, high-contrast, and extremely high color-saturation displays (e.g., gaming laptops and car screens) will be highly competitive in the high-end display market.

## Figures and Tables

**Table 1:** Parameter settings for the simulation.

Brightness enhancement film	Polyethylene terephthalate with a refractive index of 1.56 and a vertex angle of 90°
Light guide layer	Poly(methyl methacrylate) with refractive index of 1.5
Light board	Lambertian diffusion surface with reflectance of 90%
Light source settings	Output power: 1 W Center wavelength: 450 nm Number of rays: 50 million

**Table 2:** Simulation of the light guide layer with various MLA microstructures.

Microstructure	Average Full Width at Half Maximum (Degree)
Without MLA structure	117.57°
With concave PSMLA structure	141.82°
With convex PSMLA structure	141.45°

**Table 3:** Thirteen-point uniformity measurement of a 17 in extra-thin, large-area, high-luminance backlight module.

Measurement Point	Luminance (nits)
P1	18,641
P2	18,825
P3	17,785
P4	18,160
P5	18,852
P6	17,234
P7	17,251
P8	17,079
P9	17,303
P10	17,854
P11	16,818
P12	16,546
P13	16,115
Average luminance	17,574

**Table 4:** Color gamut coordinate parameters of the extra-thin, large-area, high-luminance surface light source module.

Color Gamut	Vertex Coordinates (*x*, *y*)
R	(0.693, 0.307)
G	(0.275, 0.708)
B	(0.142, 0.038)

## Data Availability

The data presented in this study are available on request from the first and corresponding authors.
